# Prevalence of orofacial and head pain: an umbrella review of systematic reviews

**DOI:** 10.22514/jofph.2024.022

**Published:** 2024-09-12

**Authors:** André Luís Porporatti, Ângela Graciela Deliga Schroder, Ashley Lebel, Nathan Moreau, Charlotte Guillouet, José Stechman-Neto, Yves Boucher

**Affiliations:** ^1^Laboratory of Orofacial NeuroBiology (EA 7543), Faculty of Dentistry, Paris-Cité University, 92120 Paris, France; ^2^NARSM (Systematic Review and Meta-analysis Center), Tuiuti University of Paraná-UTP, 82.010-210 Curitiba, PR, Brazil; ^3^Pitié Salpêtrière Hospital (AP-HP), 75013 Paris, France; ^4^Bretonneau Hospital (AP-HP), 75018 Paris, France; ^5^Rothschild Hospital (AP-HP), 75012 Paris, France

**Keywords:** Prevalence, Orofacial pain, Headache, Temporomandibular disorder, Neuropathic pain, Umbrella review

## Abstract

Head pain (HP) and orofacial pain (OFP) are the most prevalent types of pain worldwide, encompassing cranial, oral and facial pain. The aim of this umbrella review was to answer the following questions: “What is the overall prevalence of HP/OFP and the different prevalences of HP/OFP conditions in adults and children?”. We searched for studies investigating the prevalence of HP/OFP in four major databases and two databases from the grey literature, based on the following PECOS inclusion criteria: (P)opulation: Adults and children; (E)xposure: Orofacial or head pain conditions such as (1) dental, periodontal and gingival, (2) temporomandibular disorders (TMD), (3) neuropathic conditions, (4) headaches, and (5) idiopathic pain conditions; (C)omparison: None; (O)utcome: Prevalence; (S)tudies: Systematic reviews and/or meta-analyses. We identified 2275 studies and after selection through eligibility criteria, 24 systematic reviews were included. The prevalence of pain in adults for different subgroups ranged from 1.12% for Burning Mouth Syndrome to 80.80% for cancer therapy-related orofacial pain. In children, it ranged from 0.20% for temporomandibular joint osteoarthrosis to 83% for all types of headache. This umbrella review based on available evidence provides integrated data illustrating the highly variable prevalence of head pain and orofacial pain both in adults and children. Considering the high specificity of head pain/orofacial pain, specific public health programs should be developed to address such highly prevalent conditions.

## 1. Introduction

Head pain (HP) and orofacial pain (OFP) are the most prevalent types of pain worldwide, encompassing cranial, oral and facial pain. Diagnosis of OFP/HP is difficult as the pain can arise from various causes, each of which possibly involving nociceptive, inflammatory, neuropathic or nociplastic mechanisms, originating from peripheral structures such as teeth, muscles, joints, mucosas or alterations in the peripheral and central nervous system, as well as neurovascular interactions [1, 2, 3]. Additionally, many patients present comorbidities that can impact these conditions, requiring the involvement of multiple medical specialists neurologist, otolaryngologist and/or OFP specialists, as well as physiotherapists and psychologists [4, 5, 6].

The complexity of the problem is further compounded by the lack of standardized language, terminology and classification among specialists, hindering diagnostic criteria agreement and impeding comprehensive research [2, 7, 8]. Establishing a widely accepted HP classification would facilitate unambiguous communication and terminology. Indeed, several classifications systems pertaining to HP/OFP have been proposed by the International Association for the Study of Pain (IASP), the American Academy of Orofacial Pain (AAOP), the International Headache Society (IHS), and other international consortia such as the Research Diagnostic Criteria for Temporomandibular Disorders (RDC/TMD and DC/TMD) or the International Classification of Orofacial Pain (ICOP) [2, 5, 6, 9, 10, 11, 12, 13, 14, 15], reflecting their respective professional interests.

Head pain and OFP differ from spinal pain and thus require specific expertise in treatment and research to enhance overall quality of care. Assessing the specific prevalence of these conditions is crucial for estimating socioeconomic costs and understanding the global and population-specific burden of OFP/HP. Approximately one-fourth of the adult population is affected by OFP/HP. Available studies report OFP prevalence ranging from 17% to 26%, with 7% to 11% classified as chronic cases [16, 17, 18].

Numerous systematic reviews focusing on the prevalence of painful orofacial conditions have been performed, particularly in the last decade. However, a comprehensive synthesis and evaluation of these reviews is still lacking. The present umbrella review thus aimed to address this issue. To the best of our knowledge, this study represents the first umbrella review addressing the prevalence of HP/OFP conditions, in adults and children, integrating population-based and clinical studies. Specifically, the aim of this study was to answer the following questions: “What is the overall prevalence of HP/OFP?” and “How does the prevalence vary between different HP/OFP conditions in adults and children?”.

## 2. Materials and methods

### 2.1 Protocol and registration

This umbrella review conformed to the Preferred Reporting Items for Systematic Reviews and Meta-Analyses (PRISMA) Checklist [19]. The protocol was registered in the international Prospective Register of Systematic Reviews (PROSPERO) under number CRD42022377910.

### 2.2 Eligibility criteria

We included systematic reviews (SR) and/or meta-analyses (MA) that evaluated the prevalence of HP or OFP conditions in adults and children. We included studies in which the painful conditions were diagnosed by validated criteria, reported by the authors. Overall, the inclusion criteria were based on the PECOS methodology [20]: Population (P): adults and children; Exposure (E): orofacial or head pain conditions, based on the ICOP classification [2], as follows (1) dental, periodontal and gingival; (2) temporomandibular disorders; (3) neuropathic conditions; (4) headaches; and (5) idiopathic pain conditions; Comparison (C): none; Outcome (O): prevalence; (S): systematic reviews and/or meta-analyses. No data, sex or language restrictions were applied to the search strategy. Based on its intrinsically painless nature, bruxism was not included in this SR.

Studies were considered as SR if they matched the following description, as proposed by the Cochrane Collaboration’s Handbook (chapter 1.2.2): “*It uses explicit, systematic methods that are selected with a view to minimizing bias, thus providing more reliable findings from which conclusions can be drawn and decisions made*”. No time or language restrictions were applied.

The exclusion criteria included: (1) Studies in animals; (2) Studies without orofacial or head pain prevalence; (3) Literature reviews, interventional studies, observational and Randomized Controled Trials (RCT) studies; letters, abstracts from conferences; case reports and personal opinions; (4) Studies that did not meet the minimum criteria to be considered a Systematic Review (risk of bias analysis missing for instance); and (5) Studies where orofacial pain was associated with (and thus indistinguishable from) another type of pain, or in a COVID sample.

### 2.3 Information sources

Detailed individual search strategies were developed in English for each bibliographic electronic database: EMBASE, PubMed (including MEDLINE), Scopus and Web of Science. A grey literature search was performed on Google Scholar and Open Grey. All database searches were conducted from the starting coverage date through 21 September 2022, and they were updated on 29 November 2023. Furthermore, the authors hand-searched the reference lists of the selected articles for any additional references that might have been missed in the database searches, and content experts in the field were contacted to suggest relevant papers.

### 2.4 Search strategy

Keywords and MeSH terms were fully explored based on a complete search strategy as follows: “systematic review” AND prevalence AND (orofacial OR dentoalveolar OR periodontal OR “temporomandibular disorder” OR “trigeminal nerve” OR “oral neuropathy” OR migraine OR “tension-type” OR headache OR “burning mouth syndrome” OR “persistent idiopathic facial pain”). Additional information on the search strategies is provided in **Supplementary Table 1 **(which can be found online). All references were managed and the duplicated hits removed with a reference manager software (EndNote X7® Basic-Thomson Reuters, New York, NY, USA).

### 2.5 Selection process

The selection process of relevant articles was conducted in two phases. In phase one, two authors (ALP and AGDS) independently evaluated the titles and abstracts of all identified electronic database citations. In phase two, the same authors evaluated the full-text data. They independently screened papers on phase one and two, applied the eligibility criteria, collected key information from the selected studies, and crosschecked the information. The final selection was based solely on full-text assessment of the studies. When disagreement appeared, a third author (YB) was involved to make a final decision about the inclusion or exclusion of studies. This selection was performed using an appropriate software (Rayyan®, Qatar Computing Research Institute, Cambridge, MA, USA).

### 2.6 Data collection process and data items

For each of the included studies, two authors (ALP and AGDS) independently collected the following items: author(s), year of publication, country, journal published, orofacial pain subgroup, diagnostic criteria used, databases searched and search date, design of included primary studies, risk of bias assessment tools, total number of articles included for SR or MA, total number of subjects, and main prevalence with 95% Confidence Interval (CI). When the required data were not complete, the reviewers (ALP and AGDS) attempted to contact the study authors to retrieve any unpublished information. Three attempts were made in a 30-day period, by email sent to the first, second and last author.

### 2.7 Study risk of bias assessment

The methodological quality of the included SR and MA were evaluated through Assessing the Methodological Quality of Systematic Reviews (AMSTAR 2), a critical appraisal tool for systematic reviews that include randomized and non-randomized studies. Decisions about scoring were agreed upon by all reviewers before beginning critical appraisal. The same two reviewers (ALP and AGDS) worked out any differences regarding data analysis. A third author (YB) was involved to steer decisions in case of uncertainty. Following these answers, overall confidence in the results of the review was rated as follows: (1) High Confidence: no critical weakness; (2) Moderate Confidence: more than one non-critical weakness; (3) Low Confidence: one critical flaw with or without non-critical weaknesses; and (4) Critically Low Confidence: more than one critical flaw with or without non-critical weaknesses. Figures illustrating the quality assessment of all included studies were generated with Review Manager 5.3 (RevMan 5.3, The Nordic Cochrane Centre, Copenhagen, Denmark) [21].

### 2.8 Effect measures

Prevalence factors measured in percentage (%) or prevalence ratio (PR), with or without 95% CI, were considered in our review.

### 2.9 Synthesis methods

Statistical pooling of data using meta-analysis could be carried out whenever studies were considered combinable and relatively homogeneous in relation to design, interventions and outcomes. Heterogeneity within studies was evaluated either by considering clinical (differences about participants, type of interventions and results), methodological (design, and risk of bias) and statistical characteristics (effect of studies) or by using the inconsistency index (*I*^2^) statistical test [21]. We also considered generating a funnel plot as a graphic to address reporting biases, if necessary.

### 2.10 Reporting bias assessment

Methodological and statistical heterogeneity were evaluated by comparing the variability in study designs and the risk of bias. Furthermore, we also assessed the risk of bias due to missing results.

## 3. Results

### 3.1 Study selection

The initial database searches, till 21 September 2022, identified 2275 studies. In addition, 100+ studies were found with Google Scholar, and none with OpenGrey. Of these, 17 from Google Scholar were selected for full-text reading. Eight additional studies were selected following hand-searching of the reference lists of the included studies, and no further study was included based on suggestion by experts. The search was updated on 29 November 2023, with a total of 844 additional papers of which 8 were included. After eliminating duplicated hits, 952 studies remained of which 854 were excluded after title and abstract review, resulting in 98 articles for phase two. During this phase, 74 of the 98 studies were excluded (reasons for exclusion are given in **Supplementary Table 2**), leaving 24 studies for qualitative synthesis. A flowchart of the process of identification, inclusion and exclusion of studies is shown in Fig. 1.

**Fig. 1.** The main sensory innervation of the head related to pain is provided by the trigeminal nerves (5th pair of cranial nerves, V) and the upper cervical plexus (C2, C3).

### 3.2 Study characteristics and results of individual studies

In the 24 SR evaluated, the total number of included articles ranged from 3 [22] to 82 [23], with a total of 734 included studies and a mean of 25 ± 22.5 studies per SR. The number of subjects in the included articles ranged from 13 to 5,980,987, with a total approximately of 7,315,559 (**Supplementary Table 3**). Five studies were conducted in Brazil; one in Brazil and the USA; one in Canada; one in China; one in Denmark and the USA; one in India; two in Iran; one in Italy; one in Italy, the UK, Czech Republic, Poland, Belgium, the Netherlands, Turkey and Iran; three in Saudi Arabia; one in Sweden; Three in the UK; one in the UK, Germany and Sri Lanka; and two in the USA.

All studies were published in English, between 2001 [24] and 2023. Fourteen studies presented prevalence data in adults, 8 in children and two SR presented data for both. Most SR did not present data separated by sex nor by age.

Included SR searched for articles on at least two databases, of which PubMed (including MEDLINE) and Embase were the most recurrent. Databases searched included: China National Knowledge Infraestructure (CNKI), CINAHL, Cochrane, EbscoHost, Embase, Global Health Data Exchange (GHDx), Google Scholar, Iranian database, IranMedex, LILACS, LIVIVO, MagIran, MEDLINE, National guidelines for adult dentistry in Sweden, Ovid, PsycINFO, PubMed, Science Direct, Scientific Information Databank, Scopus, Wanfang, Web of Science, World Health Organization (WHO), Global Index Medicus and Wiley Online Library.

Studies encompassed different orofacial conditions and were separated by groups: Burning Mouth Syndrome (BMS), cancer-related orofacial pain, chronic orofacial pain, dental pain, headache, neuropathic pain and TMD.

One SR presented the prevalence of BMS in adults using the clinical diagnostic criteria and by IASP [25]. No SR in BMS was performed in children. Two SR on cancer-related orofacial pain in adults, pain was often reported using quality of life questionnaires or clinical examination [23, 26]. No SR in cancer-related orofacial pain was performed in children. For chronic orofacial pain, two SR presented data for children, one used the IASP guidelines and International Classification of Diseases (ICD-11) for diagnostic criteria [27], and in another the diagnostic criteria were not described [28]. Regarding dental pain, one SR in adults evaluated the prevalence of dentin hypersensitivity in adults by means of clinical exam, questionnaire and thermal tests [29]; and one SR evaluated toothache in children by means of self-report/parental report, clinical records, and visual analog scales [30] For SR of prevalence of headache, six studies were performed in adults using the definition of Blau, 1984, ID Migraine test, ICHD, IHS [31, 32, 33, 34, 35, 36], and five in children using IHS, ICHD, IASP, ICD-11 [27, 28, 37, 38, 39]. For SR of neuropathic pain, 2 SR in adults performed diagnosis by means of ICHD/IHS [22, 40]; and no SR in neuropathic pain was founded in children. Three SR in adults [41, 42, 43] and four in children [41, 43, 44, 45] performed the prevalence of TMD and the diagnostic criteria used were the RDC/TMD and DC/TMD.

Six studies were population-based studies, four clinical-based studies and 14 included both. Different types of study designs were included: case series, case-control studies, clinical trials, cohort studies, comparative studies, controlled before and after, cross-sectional, descriptive case-only design, epidemiological studies, longitudinal, non-comparative, non-randomized, observational, pilot, population-based studies, prospective, randomized clinical trials, and retrospective studies.

### 3.3 Risk of bias in studies

One study was classified as having low overall confidence in the results and 23 as moderate confidence. The high risk of bias was related to critical weakness in the performance of a comprehensive literature search strategy, a satisfactory technique for assessing the risk of bias, and a satisfactory explanation for, and discussion of, any heterogeneity, accounting for risk of bias. One important point is that most of the SR had not reported the sources of funding for the studies included in the review (non-critical weakness). The complete item list is presented in Fig. 2 and **Supplementary Table 4**.

**Fig. 2.** Flow diagram of literature search and selection criteria (adapted from PRISMA).

### 3.4 Results of syntheses

Based on data heterogeneity presented in the included studies in which results were derived from different types of orofacial conditions, we could not calculate the overall prevalence of HP/OFP in Adults and Children and no meta-analysis was performed. A descriptive analysis can be found in Table 1.

**Table 1.** Overall results.

Adults Subgroup	Pain Diagnosis	Author	Study Design	Number of Articles	Total Sample	Prevalence (in %)	Risk of Bias (Level of Confidence)
BMS							
	BMS in clinical patients	Wu S, 2021	C	18	86,591	7.72	MODERATE
	BMS in general population	Wu S, 2021	P	18	5861	1.15	MODERATE
	BMS in female general population	Wu S, 2021	P	18	26,632	1.73	MODERATE
	BMS in male general population	Wu S, 2021	P	18	5641	0.38	MODERATE
Cancer							
	Orofacial Pain prior to Cancer therapy	Epstein J, 2010	C	39	NR	49.50	MODERATE
	Orofacial Pain during Cancer therapy	Epstein J, 2010	C	39	NR	80.80	MODERATE
	Orofacial Pain at the end of Cancer therapy	Epstein J, 2010	C	39	NR	69.70	MODERATE
	Orofacial Pain 6-months post Cancer therapy	Epstein J, 2010	C	39	NR	36.20	MODERATE
	Head and Neck Cancer Pain After Treatment	Macfarlane TV, 2012	C & P	82	3112	41.50	MODERATE
	Head and Neck Cancer Pain Before Treatment	Macfarlane TV, 2012	C & P	82	1334	56.80	MODERATE
Dental							
	Dentin Hypersensitivity	Favaro Zeola L, 2018	C & P	65	97,845	33.50	MODERATE
Headache							
	Hemicrania Continua	Al-Khazali H, 2023	C	11	9854	1.78	MODERATE
	Migraine in Saudi Arabia	Albalawi M, 2023	C & P	36	55,061	0.23	LOW
	Headache in India	Dhiman V, 2021	C & P	6	16,316	6.47	MODERATE
	Migraine in Arab Countries	El-metwally A, 2020	P	23	222 to 33,000	2.60–32.00	MODERATE
	Migraine in Arab Countries	El-metwally A, 2020	C	23	222 to 33,000	7.90–78.50	MODERATE
	Migraine in Iran	Farhadi Z, 2016	C & P	30	33,873	14.00	MODERATE
	Migraine in Iran	Mohammadi P, 2023	P	10	12,534	15.10	MODERATE
Neuropathic							
	Trigeminal Neuralgia	De Toledo IP, 2016	P	3	18,715	0.03–0.30	MODERATE
	All types of Neuropathic Pain	van Hecke O, 2013	P	21	NR	3.00–17.00	MODERATE
	Postherpetic neuralgia	van Hecke O, 2013	P	21	NR	3.9–42.0/100,000 PY	MODERATE
	Trigeminal Neuralgia	van Hecke O, 2013	P	21	NR	12.6–28.9/100,000 PY	MODERATE
	Painful Diabetic Peripheral Neuropathy	van Hecke O, 2013	P	21	NR	15.3–72.3/100,000 PY	MODERATE
	Glossopharyngeal Neuralgia	van Hecke O, 2013	P	21	NR	0.2–0.4/100,000 PY	MODERATE
Other							
	Orofacial Pain in General	Macfarlane TV, 2001	P	59	NR	13.00 (1–48)	MODERATE
TMD							
	All types of TMD	Melo V, 2023	C & P	8	1258	36.10	MODERATE
	Muscular TMD	Melo V, 2023	C & P	8	5244	9.00	MODERATE
	TMD in male	Melo V, 2023	C & P	8	1912	29.30	MODERATE
	TMD in female	Melo V, 2023	C & P	8	1989	37.00	MODERATE
	DJD in Juvenile Idiopathic Arthritis	Pantoja LLQ, 2018	C	32	292	40.42 (n = 47) to 93.33 (n = 5)	MODERATE
	DJD in Rheumatoid	Pantoja LLQ, 2018	C	32	140	45.00 (n = 20) to 92.85 (n = 56)	MODERATE
	DJD in TMD	Pantoja LLQ, 2018	C	32	1472	18.01 (n = 1038) to 84.74 (n = 118)	MODERATE
	Articular TMD	Valesan LF, 2021	C & P	21	11,535	31.10	MODERATE
	DJD in TMD	Valesan LF, 2021	C & P	21	NR	9.80	MODERATE
	Arthralgia TMD	Valesan LF, 2021	C & P	21	NR	12.80	MODERATE
	Osteoarthritis TMD	Valesan LF, 2021	C & P	21	NR	1.80	MODERATE
	Osteoarthrosis TMD	Valesan LF, 2021	C & P	21	NR	15.90	MODERATE
Chronic Orofacial Pain							
	Orofacial Pain	Liao ZW, 2022	P	27	165,794	8.00	MODERATE
Dental							
	Toothache	Santos PS, 2022	C & P	70	347,496	36.20	MODERATE
Headache							
	Migraine	Abu-Arafeh I, 2010	P	37	131,228	7.70	MODERATE
	All types of Headache	Abu-Arafeh I, 2010	P	37	80,876	58.80	MODERATE
	Migraine	Asraf N, 2023	C & P	7	17,115	37–51 in 7 yo 57–82 in 15 yo	MODERATE
	All types of Headache	King S, 2011	C & P	42	29,746	8.00–83.00	MODERATE
	Migraine	King S, 2011	C & P	42	NR	3.00–10.00 (median = 8%)	MODERATE
	Tension-Type Headache	King S, 2011	C & P	42	NR	1.00–73.00 (median = 25%)	MODERATE
	Headache	Liao ZW, 2022	P	27	52,406	4.00	MODERATE
	Migraine	Onofri A, 2023	C & P	40	129,008	11.00	MODERATE
	Migraine with aura	Onofri A, 2023	C & P	40	40,775	3.00	MODERATE
	Migraine without aura	Onofri A, 2023	C & P	40	40,775	8.00	MODERATE
	Tension-Type Headache	Onofri A, 2023	C & P	40	67,089	17.00	MODERATE
TMD							
	All types of TMD	Christidis N, 2019	C & P	6	32,749	7.30–30.40	MODERATE
	Self-reported TMD-pain	Christidis N, 2019	C & P	6	NR	4.20–32.30	MODERATE
	All types of TMD	Melo V, 2023	C & P	8	2649	31.80	MODERATE
	Overall TMD using DC/TMD	Minervini G, 2023	C	3	1914	38.40	MODERATE
	TMD in female using DC/TMD	Minervini G, 2023	C	3	1093	44.70	MODERATE
	TMD in male using DC/TMD	Minervini G, 2023	C	3	821	30.00	MODERATE
	Articular TMD	Valesan LF, 2021	C & P	21	11,535	11.30	MODERATE
	Degenerative Joint Disease in TMD	Valesan LF, 2021	C & P	21	NR	0.40	MODERATE
	Arthralgia TMD	Valesan LF, 2021	C & P	21	NR	1.90	MODERATE
	Osteoarthritis TMD	Valesan LF, 2021	C & P	21	NR	0.30	MODERATE
	Osteoarthrosis TMD	Valesan LF, 2021	C & P	21	NR	0.20	MODERATE

*Legend: BMS: Burning Mouth Syndrome; C: Clinical Studies; C & P: Clinical and Populational Studies; DJD: Degenerative Joint Disease; PY: person-years; P: Populational Studies; NR: Not Reported; TMD: Temporomandibular Disorders; yo: years-old. Arab Countries are: Saudi Arabia, Egypt, Kuwait, Bahrain, Qatar, Oman, Iraq, Syria, Lebanon, Morocco, Algeria, Sudan, Libya, Tunisia and Jordan.*

### 3.5 Adults

A total of 16 studies presented prevalence data in adults. The prevalence for different subgroups ranged from 1.12% for BMS [25] to 80.80% for cancer therapy-related orofacial pain [26]; including dentin hypersensitivity with 33.50% [29]; headaches ranging from 1.78% in hemicrania continua [31] to 78.50% in migraine in Arab countries [34]; neuropathic pain ranging from 0.03% in trigeminal neuralgia [22] to 17.00% in all types of neuropathic pain [40]; 13.00% for orofacial pain in general [24]; and TMD ranging from 1.80% in temporomandibular joint osteoarthritis [43] to 36.10% in all types of TMD [41].

### 3.6 Children

A total of 10 studies presented prevalence data in children. The prevalence for different subgroups ranged from 0.2% in temporomandibular joint ﻿osteoarthrosis [43] to 83% in all types of headache [28], including 8% in chronic orofacial pain [27]; 36.20% in toothache [30]; headache ranged from 3% in migraine with aura [39] to 83% in all types of headache [28]; and TMD ranged from 0.20% in temporomandibular joint osteoarthrosis [43] to 38.40% in all types of TMD [41].

### 3.7 Risk of bias across studies and reporting biases

Overall, only moderate critical weaknesses were found in the studies included in the meta-analysis. The main problem related to reporting biases was a lack of standardization of values for 95% CI; for example, in some studies the values were only presented in main percentage and not as precise numerical values with range. No publication bias was visible in the articles. Since the number of included SR separated by groups were low, it was not possible to explore causes of publication bias across studies using a funnel plot analysis.

## 4. Discussion

### 4.1 Significance

This study is the first comprehensive umbrella review to combine available evidence on the prevalence of orofacial and head pain, both in adults and in children. This umbrella review, which included 24 systematic reviews involving individuals suffering from pain in the orofacial and/or head region, revealed a prevalence for adults ranging from 1.12% for BMS to 80.80% in cancer therapy-related orofacial pain, and for children ranging from 0.2% in temporomandibular joint osteoarthrosis [43] to 83% in all types of headache.

Several studies have emphasized the unique characteristics of head pain as compared to spinal pain, encompassing differences in anatomy, pathophysiological mechanisms, clinical features, impact on quality of life, treatment approaches and responses. The results of this meta-analysis underscore the need for research programs and public health policies targeted at specifically addressing head pain and its consequences in terms of quality of life and socioeconomic costs [46, 47, 48, 49, 50, 51, 52]. For instance, the annual direct and indirect cost per individual with persistent dentoalveolar pain has been estimated to be £27.317 in Portugal [51]. Similarly, studies on migraineurs undergoing prophylactic treatments have reported significantly higher annual costs for outpatient visits, neurology outpatient visits, emergency department visits, and hospitalizations in the prophylaxis group compared to the non-prophylaxis group [53]. Another study demonstrated that each employee with a headache disorder incurred an annual personal cost of €664.88 [49]. Unfortunately, no data related to the overall prevalence of spinal pain is available to make relevant comparisons.

In this study, different subgroups of Orofacial or Head pain were found to be more prevalent in adult patients compared to children. This finding is supported by a positive correlation between the chronicity of pain, its maturation time, patient lifespan and comorbidity factors. Children perceive pain differently than adults, primarily due to various underlying biopsychosocial determinants and the immaturity of emotional control [54, 55].

Chronic pain occurs in 19% of adult Europeans [46], and in high-income countries the estimated global prevalence of headache disorder is 52.0% [56]. Should one want to separate results from different countries or even different continents, we still have insufficient data to perform this kind of analysis. This is unfortunate, as comprehensive pain data related to specific populations and pain types are crucial for informing targeted public interventions, as suggested by Rikard *et al*. [57] 2023.

When considered as a whole, the prevalence of orofacial and head pain is almost comparable to that of other non-communicable chronic diseases such as hypertension (32% in woman, 34% in men) in 2019 [58, 59] or anemia (24.3% in 2021) [58]. In that respect, it requires proper attention and care by any and all health professionals, but also by relevant stakeholders, to advocate for the proper means to understand, prevent and treat such highly bothersome diseases. Indeed, orofacial and head pain conditions are usually considered more painful and bothersome than their spinal counterparts [58, 59]. Nevertheless, because of the high complexity and specificity of the cephalic region, HP and OFP conditions are usually managed among numerous and diverse medical specialties (neurology, ophthalmology, ENT, dentistry, oral surgery, dermatology…) and healthcare professions (medical doctors, dentists, physiotherapists…), with little to no overlap between them [60], resulting in significant diagnostic and treatment delay. One first step towards increasing such crosstalk between specialties and professions can be found in advocating for common terminology and diagnostic criteria as aforementioned. The creation of the first International Classification of Orofacial Pain is a laudable initiative in that respect [61], even if some aspects can be questioned such as the introduction of the obfuscating category “orofacial pains resembling presentations of primary headaches” instead of considering them as resulting from true primary headaches expressing their symptoms in a different territory.

### 4.2 Challenges in defining head pain and orofacial pain

While the objective of this article was to provide epidemiological data on HP and OFP, it acknowledges the challenges in properly defining these conditions. Diagnosis and classification of HP are complex due to the overlapping of anatomical structures and the functional organization of neural elements, as previously mentioned. Both cranial and facial structures are innervated by the trigeminal nerve and its three subdivisions [2, 62], leading to specific diagnostic challenges. For example, although mostly innervated by the V_1_ branch (ophthalmic nerve), the meninges also receive V_2_ (maxillary nerve) and V_3_ (mandibular nerve) innervation [63] explaining why primary headaches can be felt as toothaches [63] or why masticatory muscle pain can elicit headaches [64]. The innervation of intracranial and orofacial structures by the same trigeminal nerve divisions, along with ganglionic or central sensitization, can lead to pain spreading to extended areas and thus mislead the clinician [10]. Additionally, HP can originate from cranial nerves but may also be referred from other regions such as the cervical spine or the heart [65]. The dental pulp’s primary afferents, for instance, can extend as far as the C7 level [66, 67], explaining why intense dental pain can be perceived in the arm, and *vice versa*. Besides trigeminal innervation, the meninges also receive an innervation from the cervical nerves [68, 69]. Recent studies have demonstrated the integration of sensory signals from the head and neck conveyed by trigeminal and cervical nerves in the brainstem trigemino-cervical complex [63, 70, 71, 72, 73], making it challenging to determine the neuroanatomical distribution of pain during clinical examination. Moreover, other cranial nerves such as the VII bis, IX (*etc.*) can also elicit HP/OFP (Fig. 3) [74, 75, 76].

**Fig. 3.** AMSTAR 2, a critical appraisal tool to assessed risk of bias summary in systematic reviews. Figures generated with Review Manager 5.3 (RevMan 5.3, The Nordic Cochrane Centre, Copenhagen, Denmark). Legend: Overall confidence in the results of the review was rated based on (1) Low risk (green point): no critical weakness; (2) Moderate risk (Yellow point): more than one non-critical weakness; (3) Low (Red point): one critical flaw with or without non-critical weaknesses; and (4) Critically High risk: more than one critical flaw with or without non-critical weaknesses.

While this study did not specifically investigate cervical pain, it is important to note the coexistence of cervical pain and head/face pain. One study found significant overlap in signs and symptoms between TMD and cervical spine disorders, with an association in approximately 70% of cases [77]. Additionally, there is a high occurrence of neck pain in patients with facial/head pain. In another study, 200 female patients at a facial pain clinic were asked to mark painful sites on body sketches. Out of the individual drawings, only 37 cases (18.5%) indicated trigeminal dermatomes, while 32 cases (16%) involved combinations of trigeminal and cervical dermatomes (C2, C3 and C4) [78].

The relationship between extra-trigeminal areas and head/face pain is also supported by animal studies. For example, an animal study highlighted the direct transmission of somatosensory information from the head and face to widespread and functionally diverse areas of the central nervous system, including the dorsal horn of the spinal cord up to the C7 level [79]. We did not include neck pain in our research equation to avoid any potential confusion, as most clinically-observed cervical pain may be related to the spinal system rather than the trigeminal system. Another reason is that neck pain is not included in the new ICOP classification. However, this issue would warrant further consideration in future research and classifications.

### 4.3 Methodological considerations

Regarding risk of bias evaluation, since AMSTAR-2 was developed specifically for bias assessment of systematic reviews of randomized and non-randomized studies [67], this tool was selected over other available tools due to the extent of this umbrella review. Indeed, AMSTAR-2 was considered a more appropriate tool for a comprehensive bias assessment of all included systematic reviews of prevalence [80].

A critical factor to be noted is that even though the included SR had searched for articles on at least two databases (PubMed, MEDLINE and Embase as the most recurrent), only 33.3% of all 24 included SR (n = 8) had searched the grey literature, such as Google Scholar, Open Grey and ProQuest. Grey literature can be defined as a literature “*produced on all levels of government, academics, business and industry in print and electronic formats, but which is not controlled by commercial publishers*”. It provides data not found within commercially published literature, reducing publication bias and facilitating a more balanced view of the evidence [80].

Finally, it is important to keep in mind that data from the same primary study may potentially have been included in two or more separate systematic reviews.

### 4.4 Limitations of the study

Although the results from this umbrella review are interesting, they should nevertheless be interpreted with caution as several factors, examined below, could hamper their validity.

Despite systematic reviews being considered the most reliable form of evidence, systematic flaws or limitations in the design or conduct of a review may result in misleading or inaccurate conclusions. In addition, since they are vital in clinical decision making and resource allocation, consistent and unbiased standards are expected across systematic reviews investigating different topics and, therefore, efforts should be made to minimize or prevent potential sources of bias [81].

The results should be interpreted with caution, as some were limited by considerable between-study heterogeneity, and most studies were of low to moderate confidence in the results. Since the number of included systematic review separated by groups were low, it was not possible to explore the causes of heterogeneity across studies using meta-regression or subgroup analyses. We speculated that specific methodological differences might, in part, explain the considerable between-study heterogeneity. First, we had a very important range of samples, which could range from 13 to 5,980,987 subjects. A small sample size for a systematic review may be due to the diagnostic difficulty or rareness of the disease. Second, validated diagnostic criteria proposed by AAOP, ICHD-3, ICOP, RDC/TMD or DC/TMD and others were not always used or even described in all systematic reviews. And third, pain was usually associated with other diseases, as multiple sclerosis, ischemic stroke, widespread pain, post-traumatic disorders, epilepsy, postural tachycardia, bipolar disorders, schizophrenia or COVID-19 infection, which were excluded in this umbrella review. These may add important limitation to our results.

### 4.5 Confounding factors

Some factors that could influence pain levels have not been described, such as age and sex, general health, sleep disturbances, use of medications, and psychosocial profile [82, 83, 84, 85].

For example, it is not clear if and how the psychosocial status of the patient may alter the pain levels. Patients with anxiety may experience more negative emotional response to pain and even increased susceptibility to stress [86]. Therefore, studies related to this topic, *i.e.*, different stressful conditions related or not to anxiety or depression should be encouraged [87].

### 4.6 Recommendations for future studies

The results of the present umbrella review may not be generalizable because of the aforementioned limitations of the included studies, possible confounding factors and moderate risk of bias, with more than one non-critical weakness. Multicentric designs should be favored in order to control for culture differences. Confounding factors such as age, sex, general health, sleep disturbances, medication use, and the psychosocial profile of patients should be analyzed in future research [85, 88, 89].

## 5. Conclusions

This umbrella review found that the prevalence of pain in adults for different subgroups ranged from 1.12% for BMS to 80.80% for cancer therapy-related orofacial pain. In children, it ranged from 0.2% in ﻿temporomandibular joint osteoarthrosis to 83% for all types of headache. Such results, based on available evidence, provide integrated data illustrating the highly variable prevalence of head pain and orofacial pain both in adults and children. Considering the high specificity of head pain/orofacial pain, specific public health programs should be developed to address such highly prevalent conditions.

## Supplementary Material


