# Supplementary material for: Prevalence of orofacial and head pain: an umbrella review of systematic reviews
Source: J Oral Facial Pain Headache. 2024 Sep 12;38(3):1–14. doi: 10.22514/jofph.2024.022 (PMC11810662; doi:10.22514/jofph.2024.022)
Supplement: Supplementary file 1 [file Supplementary-material.docx]

Supplementary material

Supplementary Table 1. Database search strategy (21 September 2022, Update 29 November 2023).

| Database | Search |
| --- | --- |
| PubMed | #6 AND #7 AND #8  #8 “systematic review” [Title] OR “meta-analysis” [Title] OR “meta-analyzes” [Title] OR “meta-analyses” [Title] OR “meta analysis” [Title] OR “meta analyzes” [Title]  #7 Prevalence [Title] OR incidence [Title] OR epidemiology [Title] OR epidemiologic [Title] OR epidemiological [Title]  #6: #1 OR #2 OR #3 OR #4 OR #5  #1 Orofacial OR dentoalveolar OR Dental OR Tooth OR Teeth OR pulpal OR endodontic OR Periodontal OR Gingival OR “Oral mucosal” OR “Oral mucosa” OR “salivary gland” OR “jaw bone”  #2 “Temporomandibular disorder” OR “Temporomandibular disorders” OR “Temporomandibular dysfunction” OR “Temporomandibular dysfunctions” OR “Myofascial” OR tendonitis OR myositis OR “Temporomandibular joint” OR TMJ OR “TMJ arthritis” OR “Temporomandibular arthritis” OR “disc displacement” OR “degenerative joint disease” OR “degenerative joint diseases” OR “degenerative joint” OR “subluxation”  #3 “cranial nerves” OR “trigeminal nerve” OR “Trigeminal neuralgia” OR “trigeminal neuropathic” OR “Orofacial neuropathic” OR “Oral neuropathic” OR “Orofacial neuropathy” OR “Oral neuropathy” OR “Atypical odontalgia” OR “glossopharyngeal nerve” OR “Glossopharyngeal neuralgia” OR “Glossopharyngeal”  #4 migraine OR “Tension-type” OR “Tension type” OR Headache OR “Trigeminal autonomic” OR cluster OR “Paroxysmal hemifacial pain” OR “Short-lasting unilateral neuralgiform” OR SUNFA OR SUNCT OR SUNA OR “Neurovascular orofacial” OR “Idiopathic orofacial” OR “Paroxysmal Hemicrania”  #5 “Burning mouth syndrome” OR “Burning mouth syndrome” [MeSH Terms] OR “Burning mouth syndromes” OR “Burning mouth syndrom” OR “glossalgia” OR glossalgias OR glossodynia OR glossodynias OR glossopyrosis OR glossopyroses OR stomatodynia OR “Persistent idiopathic facial pain” OR “PIFP” OR “Persistent idiopathic dentoalveolar pain” |
| Scopus | #6 AND #7 AND #8  #8 TITLE (“systematic review” OR “meta-analysis” OR “meta-analyzes” OR “meta-analyses” OR “meta analysis” OR “meta analyzes”)  #7 TITLE (Prevalence OR incidence OR epidemiology OR epidemiologic OR epidemiological)  #6: #1 OR #2 OR #3 OR #4 OR #5  #1 Orofacial OR dentoalveolar OR Dental OR Tooth OR Teeth OR pulpal OR endodontic OR Periodontal OR Gingival OR “Oral mucosal” OR “Oral mucosa” OR “salivary gland” OR “jaw bone”  #2 “Temporomandibular disorder” OR “Temporomandibular disorders” OR “Temporomandibular dysfunction” OR “Temporomandibular dysfunctions” OR “Myofascial” OR tendonitis OR myositis OR “Temporomandibular joint” OR TMJ OR “TMJ arthritis” OR “Temporomandibular arthritis” OR “disc displacement” OR “degenerative joint disease” OR “degenerative joint diseases” OR “degenerative joint” OR “subluxation”  #3 “cranial nerves” OR “trigeminal nerve” OR “Trigeminal neuralgia” OR “trigeminal neuropathic” OR “Orofacial neuropathic” OR “Oral neuropathic” OR “Orofacial neuropathy” OR “Oral neuropathy” OR “Atypical odontalgia” OR “glossopharyngeal nerve” OR “Glossopharyngeal neuralgia” OR “Glossopharyngeal”  #4 migraine OR “Tension-type” OR “Tension type” OR Headache OR “Trigeminal autonomic” OR cluster OR “Paroxysmal hemifacial pain” OR “Short-lasting unilateral neuralgiform” OR SUNFA OR SUNCT OR SUNA OR “Neurovascular orofacial” OR “Idiopathic orofacial” OR “Paroxysmal Hemicrania”  #5 “Burning mouth syndrome” OR “Burning mouth syndromes” OR “Burning mouth syndrom” OR “glossalgia” OR glossalgias OR glossodynia OR glossodynias OR glossopyrosis OR glossopyroses OR stomatodynia OR “Persistent idiopathic facial pain” OR “PIFP” OR “Persistent idiopathic dentoalveolar pain” |
| Web of Science | #6 AND #7 AND #8  #8 TITLE (“systematic review” OR “meta-analysis” OR “meta-analyzes” OR “meta-analyses” OR “meta analysis” OR “meta analyzes”)  #7 TITLE (Prevalence OR incidence OR epidemiology OR epidemiologic OR epidemiological)  #6: #1 OR #2 OR #3 OR #4 OR #5  #1 Orofacial OR dentoalveolar OR Dental OR Tooth OR Teeth OR pulpal OR endodontic OR Periodontal OR Gingival OR “Oral mucosal” OR “Oral mucosa” OR “salivary gland” OR “jaw bone”  #2 “Temporomandibular disorder” OR “Temporomandibular disorders” OR “Temporomandibular dysfunction” OR “Temporomandibular dysfunctions” OR “Myofascial” OR tendonitis OR myositis OR “Temporomandibular joint” OR TMJ OR “TMJ arthritis” OR “Temporomandibular arthritis” OR “disc displacement” OR “degenerative joint disease” OR “degenerative joint diseases” OR “degenerative joint” OR “subluxation”  #3 “cranial nerves” OR “trigeminal nerve” OR “Trigeminal neuralgia” OR “trigeminal neuropathic” OR “Orofacial neuropathic” OR “Oral neuropathic” OR “Orofacial neuropathy” OR “Oral neuropathy” OR “Atypical odontalgia” OR “glossopharyngeal nerve” OR “Glossopharyngeal neuralgia” OR “Glossopharyngeal”  #4 migraine OR “Tension-type” OR “Tension type” OR Headache OR “Trigeminal autonomic” OR cluster OR “Paroxysmal hemifacial pain” OR “Short-lasting unilateral neuralgiform” OR SUNFA OR SUNCT OR SUNA OR “Neurovascular orofacial” OR “Idiopathic orofacial” OR “Paroxysmal Hemicrania”  #5 “Burning mouth syndrome” OR “Burning mouth syndromes” OR “Burning mouth syndrom” OR “glossalgia” OR glossalgias OR glossodynia OR glossodynias OR glossopyrosis OR glossopyroses OR stomatodynia OR “Persistent idiopathic facial pain” OR “PIFP” OR “Persistent idiopathic dentoalveolar pain” |
| EMBASE | #6 AND #7 AND #8  #8: ti Prevalence OR incidence OR epidemiology OR epidemiologic OR epidemiological  #7: ti systematic review OR meta-analysis OR meta-analyzes OR meta-analyses OR meta analysis OR meta analyzes  #6: #1 OR #2 OR #3 OR #4 OR #5  #1 Burning mouth syndrome OR Burning mouth syndromes OR Burning mouth syndrom OR glossalgia OR glossalgias OR glossodynia OR glossodynias OR glossopyrosis OR glossopyroses OR stomatodynia OR Persistent idiopathic facial pain OR PIFP OR Persistent idiopathic dentoalveolar pain  #2 migraine OR Tension-type OR Tension type OR Headache OR Trigeminal autonomic OR cluster OR Paroxysmal hemifacial pain OR Short-lasting unilateral neuralgiform OR SUNFA OR SUNCT OR SUNA OR Neurovascular orofacial OR Idiopathic orofacial OR Paroxysmal Hemicrania  #3 cranial nerves OR trigeminal nerve OR Trigeminal neuralgia OR trigeminal neuropathic OR Orofacial neuropathic OR Oral neuropathic OR Orofacial neuropathy OR Oral neuropathy OR Atypical odontalgia OR glossopharyngeal nerve OR Glossopharyngeal neuralgia OR Glossopharyngeal  #4 Temporomandibular disorder OR Temporomandibular disorders OR Temporomandibular dysfunction OR Temporomandibular dysfunctions OR Myofascial OR tendonitis OR myositis OR Temporomandibular joint OR TMJ OR TMJ arthritis OR Temporomandibular arthritis OR disc displacement OR degenerative joint disease OR degenerative joint diseases OR degenerative joint OR subluxation  #5 Orofacial OR dentoalveolar OR Dental OR Tooth OR Teeth OR pulpal OR endodontic OR Periodontal OR Gingival OR Oral mucosal OR Oral mucosa OR salivary gland OR jaw bone |
| Google Scholar | (Prevalence OR incidence OR epidemiology OR epidemiologic OR epidemiological) AND (systematic review OR meta-analysis OR meta-analyzes OR meta-analyses OR meta analysis OR meta analyzes) AND (Burning mouth syndrome OR Burning mouth syndromes OR Burning mouth syndrom OR glossalgia OR glossalgias OR glossodynia OR glossodynias OR glossopyrosis OR glossopyroses OR stomatodynia OR Persistent idiopathic facial pain OR PIFP OR Persistent idiopathic dentoalveolar pain)  (Prevalence OR incidence OR epidemiology OR epidemiologic OR epidemiological) AND (systematic review OR meta-analysis OR meta-analyzes OR meta-analyses OR meta analysis OR meta analyzes) AND (migraine OR Tension-type OR Tension type OR Headache OR Trigeminal autonomic OR cluster OR Paroxysmal hemifacial pain OR Short-lasting unilateral neuralgiform OR SUNFA OR SUNCT OR SUNA OR Neurovascular orofacial OR Idiopathic orofacial OR Paroxysmal Hemicrania)  (Prevalence OR incidence OR epidemiology OR epidemiologic OR epidemiological) AND (systematic review OR meta-analysis OR meta-analyzes OR meta-analyses OR meta analysis OR meta analyzes) AND (cranial nerves OR trigeminal nerve OR Trigeminal neuralgia OR trigeminal neuropathic OR Orofacial neuropathic OR Oral neuropathic OR Orofacial neuropathy OR Oral neuropathy OR Atypical odontalgia OR glossopharyngeal nerve OR Glossopharyngeal neuralgia OR Glossopharyngeal)  (Prevalence OR incidence OR epidemiology OR epidemiologic OR epidemiological) AND (systematic review OR meta-analysis OR meta-analyzes OR meta-analyses OR meta analysis OR meta analyzes) AND (Temporomandibular disorder OR Temporomandibular disorders OR Temporomandibular dysfunction OR Temporomandibular dysfunctions OR Myofascial OR tendonitis OR myositis OR Temporomandibular joint OR TMJ OR TMJ arthritis OR Temporomandibular arthritis OR disc displacement OR degenerative joint disease OR degenerative joint diseases OR degenerative joint OR subluxation)  (Prevalence OR incidence OR epidemiology OR epidemiologic OR epidemiological) AND (systematic review OR meta-analysis OR meta-analyzes OR meta-analyses OR meta analysis OR meta analyzes) AND (Orofacial OR dentoalveolar OR Dental OR Tooth OR Teeth OR pulpal OR endodontic OR Periodontal OR Gingival OR Oral mucosal OR Oral mucosa OR salivary gland OR jaw bone) |
| OpenGrey | Prevalence AND Orofacial |

Supplementary Table 2. Articles excluded and the reasons for exclusion (n = 74).

| Author year | Reasons for exclusion* |
| --- | --- |
| Al-Khazali HM, 2022 | 5 |
| Ayouni I, 2019 | 2 |
| Campos LGL, 2021 | 2 |
| Chai NC, 2014 | 4 |
| Chekol B, 2021 | 5 |
| Conti Reus J, 2021 | 2 |
| Da Silva, 2016 | 2 |
| De Kanter RJAM, 1993 | 4 |
| De Oliveira Souza, 2020 | 5 |
| De Toledo IP, 2016 b | 5 |
| Do Nascimento L, 2020 | 5 |
| Duko B, 2020 | 5 |
| Eigenbrodt A, 2022 | 5 |
| Fayaz A, 2016 | 2 |
| Fischera M, 2007 | 4 |
| Fishbain DA, 2016 | 5 |
| Foley PL, 2012 | 5 |
| Fornaro M, 2015 | 5 |
| Gholami A, 2020 | 4 |
| Haggman-Henrikson B, 2014 | 5 |
| Hara ES, 2012 | 2 |
| Harriott A, 2020 | 5 |
| Houshi S, 2022 | 5 |
| Islam MA, 2019 | 5 |
| Jackson T, 2015 | 4 |
| Jackson T, 2016 | 4 |
| Kleykamp BA, 2020 | 5 |
| Kleykamp BA, 2022 | 5 |
| Lai YC, 2020 | 5 |
| Leo R, 2016 | 5 |
| Lietz J, 2018 | 2 |
| Manfredini D, 2011 | 4 |
| Manfredini D, 2016 | 2 e 5 |
| Mansfield K, 2016 | 2 |
| Minervini G, 2022 | 2 |
| Miranda VS, 2012 | 2 |
| Mirmosayyeb O, 2020 | 5 |
| Mnguni N, 2021 | 3 |
| Moisset X, 2016 | 5 |
| Murray C, 2022 | 2 |
| Mutiawati E, 2021 | 5 |
| Nampiaparampil DE, 2008 | 4 |
| Narouze S, 2015 | 2 |
| Natoli JL, 2009 | 4 |
| Norton J, 2020 | 5 |
| O’Connor A, 2007 | 4 |
| O’Connor E, 2020 | 2 |
| Pak JG, 2010 | 2 |
| Ray JC, 2022 | 5 |
| Ribeiro DC, 2018 | 2 |
| Rotter G, 2019 | 2 |
| Ryan J, 2019 | 2 |
| Sa K, 2019 | 2 |
| Santos B, 2017 | 5 |
| Sathorn C, 2007 | 2 |
| Schurks M, 2014 | 5 |
| Siqveland J, 2017 | 5 |
| Sharma P, 2022 | 2 |
| Shaw L, 2017 | 4 |
| Silva MAG, 2020 | 2 |
| Simmonds M, 2016 | 3 |
| Stewart WF, 1995 | 4 |
| Stubbs B, 2014 | 5 |
| Stubbs B, 2014 | 5 |
| Van den Beuken–Van ever Dingen MHJ, 2016 | 2 |
| Vishwanathaiah S, 2021 | 2 |
| Turki A, 2022 | 2 |
| Wang X, 2015 | 5 |
| Westergaard ML, 2013 | 4 |
| Woldeamanuel YM, 2014 | 4 |
| Woldeamanuel YW, 2017 | 4 |
| Xie C, 2018 | 5 |
| Xie Q, 2022 | 5 |
| Yakkaphan P, 2022 | 5 |

Legend: (1) Studies in animals; (2) Studies without orofacial Pain Prevalence; (3) Literature reviews, intervention studies, observational and RCT studies; letters, abstracts from conferences; case reports and personal opinions; (4) studies that did not meet the minimum criteria for Systematic Review (risk of bias analysis missing for instance); and (5) Studies where orofacial pain was associated with (and thus indistinguishable from) another type of pain, or in a COVID sample.

Supplementary Table 3. Summary of descriptive characteristics of included articles (n = 24).

| Author  Year  Country  Journal | Subgroup | Diagnostic Criteria | Databases searched (Search date) | Included primary studies | Study Design | Risk of bias assessment tools | Total number of Articles included | Total number of patients  Gender  Age | Main Prevalence |
| --- | --- | --- | --- | --- | --- | --- | --- | --- | --- |
| Abu-Arafeh I  2010  UK  Developmental Medicine & Child Neurology | Headache  Children (under 20 y.o) | IHS 1988 or 2004 | PubMed  Cochrane  Embase  Google Scholar  (31 December 2007) | Population based studies of randomized selected participants | 1 (Population based) | Criteria applied by the authors | 50 Quali (37 MA) | 80,876 children  44,988 F—55.62%  3–20 years  Pain >6 months | The overall calculated prevalence of headache (at any point in time) is 58.4% with a 95% CI (58.1–58.8%).  The OR for prevalence of headache in females compared with males is 1.53 (1.48–1.60).  Overall prevalence of migraine in children and adolescents is 7.7% (95% CI 7.6–7.8). |
| Albalawi M  2023  Saudi Arabia  Cureus | Migraine  Adults | All | PubMed  Cochrane  Google Scholar  Web of Science  Ovid  NR | Cross-sectional, case controls and community-based surveys | 1 and 2 | ROB and Egger | 36 Quali and MA | 55,061  >15 years  NA | Pooled proportion of migraine among all selected studies was 0.225617 (95% CI = 0.172749 to 0.28326). |
| Al-Khazali HM  2023  Denmark and USA  Cephalalgia | Hemicrania continua | ICHD-2, ICHD-3b  ICHD-3 | PubMed  Embase  (18/07/2022) | 6 prospective  5 retrospective cohort | 2 (Clinical based) | JBI Critical Appraisal Checklist for Studies Reporting Prevalence Data | 11 Quali and MA | 9854  NA | The pooled relative frequency of hemicrania continua was estimated to be 1.8% (95% CI, 1.0–3.3) among adult patients who were evaluated for headache in a clinic-based setting.  The three most common symptoms associated with hemicrania continua were lacrimation (72.3%), conjunctival injection (69.8%), and restlessness/agitation (60.2%). |
| Asraf N  2023  Saudi Arabia  International Journal of Life science and Pharma Research | Migraine  Children | ICHD-3  IHS  IHS2  Questionnaire | PubMed/MEDLINE  Scopus  Embase  EbscoHost  Google Scholar  From October 2002 to October 2022 | Cross-sectional, Cohort and community-based surveys | 1 and 2 | ROBINS-I | 7 Quali | 17,115  4–18  50% F | Prevalence of headaches increases with age, from 37–51% in 7 year-old to 57–82% in 15 year-old. |
| Christidis N  2019  Sweden  Journal of Oral Rehabilitation | TMD  Children (under 19 y.o) | RDC/TMD  DC/TMD | PubMed  Web of Science  National guidelines for adult dentistry in Sweden | Cross-sectional | 1 and 2 | Checklist for the Methodological evaluation of Observational Research (MORE) | 6 Quali and MA | 32,749  156,91 F—47.91%  10–19 years | Prevalence of TMD seems to have a range from 7.3% up to 30.4%. In three of the studies, the most common TMD-diagnosis was myofascial pain. In the other two the most common TMD diagnosis was disc displacement with reduction. |
| De Toledo IP  2016  Brazil  Journal of American Dental Association | Trigeminal Neuralgia | ICHD-3/IHS | LILACS  PubMed  Science Direct  Scopus  Web of Science  Google Scholar  (08 May 2015) | Cross-sectional | 1 | Agency for Healthcare Research and Quality (AHRQ) | 3 Quali | 18,715  16 cases–12 F (75%)  18 to >65 years | Higher prevalence (range, 0.03–0.3%) in women older than 40 years that usually affected the maxillary and mandibular branches |
| Dhiman V  2021  India  Neurology India | Headache in India | Different case definitions were adopted for inclusion of studies. In addition, we also included studies that did not clearly report the definition criteria but were done on large representative samples. | PubMed  Global Health Data Exchange (GHDx) Institute of Health Metrics and Evaluation (IHME)  Google Scholar  (July 2019) | Cross-sectional studies and community-based surveys | 1 and 2 | JBI Critical Appraisal Checklist for Studies Reporting Prevalence Data | 6 Quali | 16,316  NA  11 to 65 years | The reporting of prevalence of headache in India has been very wide, ranging from as low as 0.2% up to 58% mainly due to varied criteria used to define different types of headaches. The pooled prevalence of headache in the present study was found to be 438.8 per 1000 population (95% CI: 287.6–602.3), which is higher than previously reported rates. |
| El-metwally A  2020  Saudi Arabia  The Scientific World Journal | Migraine in Arab Countries | The definition of Blau, 1984.  IHS  ICD-2  ID Migraine test  WHO | PubMed  Embase  (from 1990 to 2019) | Cross-sectional | 1 and 2 | Newcastle-Ottawa Scale | 23 Quali and MA | Sample size ranged from 222 to >33,000  Total 5,980,987  >6 year-old, most of the participants were at least 18 year-old. | Migraine prevalence among the general population was estimated in ten articles and showed a range between 2.6% and 32%, while four other studies were performed on clinic attendees and showed a prevalence ranging from 7.9% to 78.5%.  Five other studies indicated that the prevalence of migraines among school children (aged 6 to 18) ranged from 7.1% to 13.7%.  Three studies also estimated prevalence among medical university students between 12.2% and 27.9%. |
| Epstein J  2010  USA  Support Care Cancer | Cancer therapy-related orofacial pain | Pain was often reported using quality of life questionnaires | MEDLINE  PubMed  EMBASE  (31 December 2008) | 33 observational studies  6 clinical trials  3 RCT  1 Non-randomized  2 controlled Before and after  21 Cohort  2 Case–control  10 Cross sectional | 2 | The quality of selected articles was assessed and scored with respect to sources of bias, representativeness, scale validity, and sample size. | 39 Quali | Not Described  NA | Pain in the Head and Neck region was found in approximately half of patients prior to cancer therapy, 81% during therapy, 70% at the end of therapy and still 36% at 6 months post-treatment. |
| Farhadi Z  2016  Iran  Iran Red Crescent Med J. | Migraine in Iran | ICHD-1  ICHD-2  ID Migraine | PubMed  Web of Science  Embase  Scopus  Ovid  Google Scholar  MagIran  IranMedex  Scientific Information Databank  (November 2015) | Cross-sectional | 1 and 2 | STROBE | 30 Quali and MA | 33,873  NA | The pooled prevalence of migraine, using the random-effects model in Iran, was 14% (95% CI 12% to17%). |
| Favaro Zeola L  2018  Brazil and USA  Journal of Dentistry | Dentin hypersensitivity | Clinical exam  Questionnaire only  Questionnaire and clinical exam for those positive to self-report  questionnaire  Stimuli during clinical exam  Thermo-evaporative only Thermo-evaporative and Tactile Thermo-evaporative and Thermal Tactile only  Thermal only  Tactile and Thermal  None (self-reported questionnaire only) | PubMed  Cochrane Library  Wiley Online Library  Web of Science (June 2018) | Cross-sectional | 1 and 2 | Newcastle-Ottawa Scale | 65 Quali and MA | 97.845  NA  mostly adults (65%) | The prevalence range was observed to be as low as 1.3% and as high as 92.1%.  The best estimate of dentin hypersensitivity was 11.5% (95% CI: 11.3%–11.7%) and the average from all studies was 33.5% (95% CI: 30.2%–36.7%). |
| King S  2011  Canada  Pain | Chronic orofacial pain in children and adolescents  (headache) | Not described | EMBASE  Medline  CINAHL  PsycINFO  (2009) | Observational | 1 and 2 | STROBE | 42 Quali and MA | 29,746  NA  Age ranged 5 to 18 years | Prevalence rates ranged substantially, and were as follows: headache: 8–83%; abdominal pain: 4–53%; back pain: 14–24%; musculoskeletal pain: 4–40%; multiple pains: 4–49%; other pains: 5–88%.  Headache  Three studies investigated prevalence rates over the past month or 3 months with prevalence rates ranging from 26% to 69% in children aged 7 to 16 years (median = 47%).  Weekly headache prevalence rates in children aged between 7 and 18 years of age were reported in 8 studies. Across these studies, prevalence of weekly headache ranged from 6% to 31% (median = 23%).  Finally, 2 studies presented prevalence rates for daily headache in children aged between 10 and 18 years with rates ranging from 1% to 9% (median = 5.1%).  Four headache studies reported prevalence rates for migraine and 3 reported prevalence rates for tension-type headaches. Prevalence rates for migraine with and without aura ranged from 3% to 10% (median = 8%), whereas prevalence rates for tension-type headache ranged much more widely, from 1% to 73% (median = 25%). |
| Liao ZW  2022  USA  eClinicalMedicine | Chronic orofacial pain  Pediatric  headaches | International Association for the Study of Pain (IASP) guidelines and International Classification of Diseases (ICD-11) | MEDLINE (*via* PubMed)  Embase (OvidSP)  CINAHL (EBSCOhost)  PsycINFO (ProQuest)  Web of Science (Clarivate)  Cochrane (Wiley)  WHO Global Index Medicus  (January 2022) | 24 Cross-Sectional  2 Cohort  1 Case-control | 1 | A quality assessment checklist for prevalence studies adapted from Hoy *et al*. [1] and published by Nguyen | 27 Quali and MA | 165,794  Ranged from 209 to 8701  72884 F (51.5%)  68642 male | Headaches were the most common study focus (N = 10), followed by musculoskeletal pain (N = 7), abdominal pain (N = 6), general/multi-site/any pain (N = 3), fibromyalgia (N = 3), and temporomandibular disorder (N = 1).  The prevalence of chronic pain in included studies has a pooled mean of 8% (95% CI 6−10). The prevalence rate for each type of chronic pain is estimated as follows: general/multi-site/any pain 20% (95% CI 16−25), MSK/back pain 9% (95% CI 7−13), abdominal pain 7% (95% CI 5−10), headache 4% (95% CI 2−10), and fibromyalgia 3% (95% CI 1−10). Only one study on chronic temporomandibular disorder pain met inclusion criteria, reporting a prevalence of 15%. |
| Macfarlane TV  2001  UK  Journal of Dentistry | Orofacial pain | Orofacial pain was self-reported and/or confirmed by dental examination. | MEDLINE (1966 ± 1998)  EMBASE (1980 ± 1998) CINAHL (1982 ± 1998)  Scientific citation index *via* BIDS ISI (1981 ± 1998)  Health CD (1990 ± 1998) | 46 Cross-Sectional  12 Cohort  1 Case-Control | 1 | Modification of the checklists designed by Downs and Blac and Crombie | 59 Quali | Not Described | The minimum prevalence of orofacial pain was 1% (current cheek pain) and the maximum was 48% (current oral or facial pain). The median prevalence of OFP was 13%. Sometimes the definition included headache.  The minimum prevalence of pain around the ear was 4% (current pain in or near the ear) and the maximum 15% (current pain in or in front of the ear). The lowest prevalence of burning tongue or mouth was reported by Lipton *et al*. [31] as 0.7% (burning mouth, past 6 months) and the highest 15% (prolonged burning sensation on oral mucosa) |
| Macfarlane TV  2012  UK  Germany and Siri lank  Journal of Oral Maxillofacial Research | Head and Neck cancer pain | Cancers of the oral mucosa, larynx, oropharynx and hypopharynx | MEDLINE  EMBASE  CINAHL  (December 2021) | 27 Cross sectional  15 Prospective  06 Retrospective  03 Retrospective chart review  02 Pilot  02 Cohort  01 Case-Control  01 Case Series  01 Follow up  01 Survey  01 Univariate and correlational descriptive  27 Study design not reported in methods | 1 and 2 | Checklists developed by Downs and Black, Crombie and Vandenbroucke *et al.* [2] | 82 Quali and MA | Minimum 13 and maximum 1761  Median: 80 (44, 154)  1334 Before Treatment  3112 After Treatment  NA | Combined estimate of pain prevalence from meta-analysis before treatment was 57%, 95% CI 43%–70% while combined estimate from meta-analysis after treatment was 42%, 95% CI 33%–50%, based on 12 and 19 studies respectively. |
| Melo V  2023  Brazil  Cranio | TMD  Adults and Children | RDC/TMD | PubMed  EMBASE  Scopus  LILACS  NR | Cross-Sectional | 1 and 2 | Joanna Briggs Institute Critical Appraisal Instrument for Studies Reporting Prevalence Data | 11 Quali and 8 MA | 6365 (3437 females, 2733 males  and 195 unspecified) people from 11 studies were  included in the review. Sample mean age ranged from  12 to 69.5 years. | The pooled prevalence of TMD was 33.6% (95% CI 31.5–35.8; *I*^2^ = 37.2). Prevalence of TMD was higher in females 37.0% (95% CI 30.9–43.3; *I*^2^ = 81.8) than in males 29.3% (95% CI 25.2–33.6; *I*^2^ = 69.7). Adults showed a prevalence of 36.1% (95% CI 33.5–38.8; *I*^2^ = 0), while children and adolescents presented with 31.8% (95% CI 29.1–34.5; *I*^2^ = 57.7). |
| Minervini G  2023  Italy  Journal of Oral Rehabilitation | TMD  Children | DC/TMD | PubMed  Web of Science  Lilacs  (30 November 2022) | Cross-Sectional | 2 | ROB 2 | 4 Quali and 3 MA | 1914  57.1% F | TMD is an increasingly common problem in children and adolescents. Its prevalence varies between 20% and 60%.  38.4% presented TMD. Among 1093 female, 489 (44.7%) presented TMD, while 247/821 male (30%) experienced TMD. |
| Mohammadi P  2023  Iran  BMC Neurology | Migraine Iran | ICHD-2  ICHD-3  IHS  IHS2  Questionnaire | Three Iranian data-  bases, MagIran and SID, with Iranian keywords and four  international databases, Science Direct, Scopus, and ISI  Web of Science and PubMed  (November  2022) | Cross-Sectional | 1 | STROBE checklist | 22 Quali and 10 MA | 12,534  10–95 years | The prevalence of migraine in the general population was calculated based on data from 10 studies.  The prevalence of migraine in the general population of Iran was calculated as 15.1% (95% CI: 10.7–20.9). |
| Onofri A  2023  Italy, UK, Czech Republic, Poland, Belgium, Netherlands, Turkey and Iran  The Journal of Headache and Pain | Headache  Children | ICHD | Embase  Medline  Web of Science  Cochrane  Google Scholar  July 2022 | 47 Cross-Sectional  1 Case-Control | 1 and 2 | JBI Critical Appraisal | 48 Quali and 40 MA | 76,782  8–18 years  16.3 to 94.4% F | The pooled prevalence of primary headaches was 11% for migraine overall (95% CI: 9–14%), 8% for migraine without aura (MwoA) (95% CI: 5–12%), 3% for migraine with aura (MwA) (95% CI: 2–4%) and 17% for tension‐type headache (TTH) (95% CI: 12–23%). The pooled prevalence of overall primary headache in children and adolescents was 62% (95% CI: 53–70%), with prevalence in females and males of 38% (95% CI: 16–66%) and 27% (95% CI: 11–53%) respectively. |
| Pantoja, LLQ  2018  Brazil  Clinical Oral Investigation | Degenerative joint disease in TMD | RDC/TMD  DC/TMD  Clinical assessment and imaging exam confirmation | LILACS  LIVIVO  PubMed  Science Direct  SCOPUS  Web of Science  (10/01/2018) | Cross-Sectional | 2 | JBI Critical Appraisal Checklist for studies Reporting Prevalence Data | 32 Quali and MA | 3435  NA | The prevalence of DJD on juvenile idiopathic arthritis patients ranged from 40.42% (n = 47) to 93.33% (n = 15) and on rheumatoid arthritis patients from 45.00% (n = 20) to 92.85% (n = 56).  Among TMD patients, the prevalence of DJD reported according to patients ranged from 18.01% (n = 1038) to 84.74% (n = 118) and reported according to joints ranged from 17.97% (n = 178) to 77.23% (n = 224). |
| Santos PS  2022  Brazil  Clinical Oral Investigation | Toothache  dental caries  Children | Self-report/parental report, clinical records, visual analogue scale of faces, DePaQ, C-DPQ) | Medline  PubMed  Scopus  Web of Science  EMBASE  LILACS  CENTRAL  (February/2021) | 68 Cross-Sectional  3 Cohort | 1 and 2 | Fowkes and Fulton Critical Appraisal Checklist | 71 Quali (70 MA) | 347,496  Children and adolescents | The overall pooled prevalence of toothache in children and adolescents was 36.2% (95% CI: 33.0–39.42; *I*^2^: 99.72%; *p* < 0.001).  Females (OR: 1.17; 95% CI: 1.08–1.26; *I*^2^: 91%; *p* < 0.001) and children and adolescents whose caregivers had ≤8 years of schooling (OR: 1.42; 95% CI: 1.30–1.56; *I*^2^: 77%; *p* < 0.001) presented the higher risk of reporting toothache.  Dental caries experience increased the risk of reporting toothache 3.49 fold (95% CI: 2.70–4.51; *I*^2^: 92% (*p* < 0.001). |
| Valesan LF  2021  Brazil  Clinical Oral Investigation | Temporomandibular joint disorders  Adults and children | RDC/TMD  DC/TMD | PubMed  EMBASE  LILACS  Scopus  Web of Science  (22 January 2020) | Inclusion criteria consisted of cross-sectional studies that evaluated the prevalence of TMJD among general population. | 1 and 2 | JBI Critical Appraisal Checklist for Studies Reporting Prevalence Data | 21 | 11,535  52.87% F  Mean age between 7 e 75 years | Adults and elders  TMJD 31.1% (10.6–63.3)  DD 19.1% (9.4–34.9)  DJD 9.8% (2.2–34.3)  Arthralgia 12.8% (6.2–24.4)  Osteoarthritis 1.8% (0.8–3.9)  Osteoarthrosis 15.9% (1.6–68.1)  Children and adolescents  TMJD 11.3% (7.6–16.4)  DD 8.3% (5.2–13.0)  DJD 0.4% (0.2–0.9)  Arthralgia 1.9% (0.9–3.9)  Osteoarthritis 0.3% (0.1–1.0)  Osteoarthrosis 0.2% (0.0–1.0)  Furthermore, for children/adolescents are as follows: The most prevalent TMJD is DDwR for adults/elderly (25.9%) and children/adolescents (7.4%). |
| van Hecke O  2013  UK  Pain | Neuropathic pain | Not Described | MEDLINE  EMBASE  ISI Web of Science  CINAHL  (December 2012) | Epidemiological researches  Population-based studies  Observational studies | 1 | STROBE checklist | 21 Quali and MA | Not Described | We categorized comparable incidence and prevalence rates into 2 main subgroups: (1) chronic pain with neuropathic characteristics (range 3–17%), and (2) neuropathic pain associated with a specific condition, including postherpetic neuralgia (3.9–42.0/100,000 person–years (PY)), trigeminal neuralgia (12.6–28.9/100,000 PY), painful diabetic peripheral neuropathy (15.3–72.3/100,000 PY), glossopharyngeal neuralgia (0.2–0.4/100,000 PY). |
| Wu S  2021  China  Oral Diseases | Burning Mouth Syndrome | AHC  Clinical diagnosis  IASP | PubMed  Embase  Web of Science  Cochrane  China National Knowledge Infraestructure (CNKI) Wanfang  (31 January 2021) | 9 Population-based studies  9 Clinical-based studies | 1 and 2 | JBI Critical Appraisal Checklist for Studies Reporting Prevalence Data | 18 Quali and MA | 26,632 in general population + 86,591 in clinical patients  Around 52% F  NA | The overall pooled prevalence of burning mouth syndrome was 1.73% (95% CI = 0.176–0.351, n = 26,632) in general population, and 7.72% (95% CI = 0.434–0.691, n = 86,591) in clinical patients.  The subgroup analysis by continent showed that among the population-based studies the prevalence in Asia (1.05%) lower than in Europe (5.58%) and North America (1.10%).  The subgroup analysis by gender showed the prevalence of female (1.15%) was higher than male (0.38%) in general population.  The subgroup analysis by age showed the prevalence was higher for people over 50 (3.31%) than under 50 (1.92%). |

Legend: BMS: Burning Mouth Syndrome; DC: Diagnostic Criteria; DD: Disk Displacements; DJD: Degenerative Joint Disease; PDPH: Post-Dural Puncture Headache; IASP: International Association for the Study of Pain; IBS: Irritable Bowel Syndrome; ICD-10: International Statistical Classification of Diseases and Related Health Problems 10th Edition; ICHD: International Classification of Headache Disorders; IHS: International Headache Society; JBI: Joanna Briggs Institute; F: Female; MA: Meta-analysis; MS: multiple sclerosis; POTS: Postural Tachycardia Syndrome; PTSD: Post-Traumatic Stress Disorder; RDC: Research Diagnostic Criteria; RLS: Restless Legs Syndrome; STROBE: Strengthening The Reporting of OBservational studies in Epidemiology; TBI: Traumatic Brain Injuries; TMD: Temporomandibular Disorder; TN: Trigeminal Neuralgia; USA: United States of America; UK: United Kingdom; y.o: year-old; CI: Confidence Interval.

Supplementary Table 4. AMSTAR 2 Critical Appraisal Tools to assessed risk of bias in systematic reviews.

|  | Author, Year | 1 | 2 | 3 | 4 | 5 | 6 | 7 | 8 | 9 | 10 | 11 | 12 | 13 | 14 | 15 | 16 | Result (Confidence) |
| --- | --- | --- | --- | --- | --- | --- | --- | --- | --- | --- | --- | --- | --- | --- | --- | --- | --- | --- |
| 1. | Abu-Arafeh I, 2010 | Y | N | Y | PY | Y | N | N | Y | Y | Y | Y | N | Y | Y | Y | N | MODERATE |
| 2. | Albalawi M, 2023 | Y | N | Y | PY | Y | Y | N | Y | N | N | Y | N | N | Y | N | Y | LOW |
| 3. | Al-Khazali HM, 2023 | Y | N | Y | Y | Y | Y | N | Y | Y | N | Y | N | Y | Y | N | Y | MODERATE |
| 4. | Asraf N, 2023 | Y | N | Y | Y | Y | Y | N | Y | Y | N | NO MA | NO MA | Y | Y | NO MA | Y | MODERATE |
| 5. | Christidis N, 2019 | Y | N | Y | PY | Y | Y | Y | Y | Y | N | NO MA | NO MA | Y | Y | NO MA | Y | MODERATE |
| 6. | De Toledo IP, 2016 | Y | Y | Y | Y | Y | Y | Y | Y | Y | N | NO MA | NO MA | Y | Y | NO MA | Y | MODERATE |
| 7. | Dhiman V, 2021 | Y | Y | Y | PY | Y | Y | Y | Y | Y | N | Y | Y | Y | Y | Y | Y | MODERATE |
| 8. | El-metwally A, 2020 | Y | N | Y | PY | Y | Y | Y | Y | Y | N | NO MA | NO MA | Y | Y | NO MA | Y | MODERATE |
| 9. | Epstein J, 2010 | Y | N | Y | PY | Y | Y | Y | Y | Y | N | Y | N | Y | Y | N | Y | MODERATE |
| 10. | Farhadi Z, 2016 | Y | N | Y | PY | Y | Y | PY | Y | Y | N | Y | Y | Y | Y | Y | N | MODERATE |
| 11. | Favaro Zeola L, 2018 | Y | N | Y | Y | Y | Y | Y | Y | Y | N | Y | Y | Y | Y | N | Y | MODERATE |
| 12. | King S, 2011 | Y | N | Y | PY | Y | Y | PY | Y | Y | N | NO MA | NO MA | Y | Y | NO MA | Y | MODERATE |
| 13. | Liao ZW, 2022 | Y | Y | Y | Y | Y | Y | Y | Y | Y | N | Y | Y | Y | Y | Y | Y | MODERATE |
| 14. | Macfarlane TV, 2001 | Y | N | Y | Y | Y | Y | PY | Y | Y | N | NO MA | NO MA | Y | Y | NO MA | N | MODERATE |
| 15. | Macfarlane TV, 2012 | Y | N | Y | Y | Y | Y | PY | Y | Y | Y | Y | N | Y | Y | N | Y | MODERATE |
| 16. | Melo V, 2023 | Y | Y | Y | Y | Y | Y | Y | Y | Y | N | Y | N | Y | Y | N | Y | MODERATE |
| 17. | Minervini, 2023 | Y | Y | Y | Y | Y | Y | Y | Y | Y | N | Y | Y | Y | Y | N | Y | MODERATE |
| 18. | Moisset X, 2016 | Y | N | Y | PY | Y | Y | Y | Y | Y | N | NO MA | NO MA | Y | Y | NO MA | Y | MODERATE |
| 19. | Mohammadi P, 2023 | Y | N | Y | Y | Y | PY | Y | Y | Y | N | Y | N | Y | Y | Y | Y | MODERATE |
| 20. | Onofri A, 2023 | Y | PY | Y | Y | Y | Y | Y | Y | Y | N | Y | Y | Y | Y | PN | Y | MODERATE |
| 21. | Pantoja LLQ, 2018 | Y | Y | Y | Y | Y | Y | Y | Y | Y | N | NO MA | NO MA | Y | Y | NO MA | Y | MODERATE |
| 22. | Santos OS, 2022 | Y | Y | Y | Y | Y | Y | N | Y | Y | N | Y | Y | Y | Y | N | Y | MODERATE |
| 23. | Valesan LF, 2021 | Y | Y | Y | Y | Y | Y | Y | Y | Y | N | Y | Y | Y | Y | N | Y | MODERATE |
| 24. | van Hecke O, 2013 | Y | N | Y | PY | Y | Y | Y | Y | Y | Y | NO MA | NO MA | Y | Y | NO MA | Y | MODERATE |
| 25. | Wu S, 2021 | Y | Y | Y | PY | Y | Y | Y | Y | Y | N | Y | Y | Y | Y | Y | Y | MODERATE |

Legend: NO MA: No meta-analysis conducted.

1.Did the research questions and inclusion criteria for the review include the components of PICO? (non-critical weakness);

2. Did the report of the review contain an explicit statement that the review methods were established prior to the conduct of the review and did the report justify any significant deviations from the protocol? (non-critical weakness);

3. Did the review authors explain their selection of the study designs for inclusion in the review? (non-critical weakness);

4. Did the review authors use a comprehensive literature search strategy? (critical weakness);

5. Did the review authors perform study selection in duplicate? (non-critical weakness);

6. Did the review authors perform data extraction in duplicate? (non-critical weakness);

7. Did the review authors provide a list of excluded studies and justify the exclusions? (non-critical weakness);

8. Did the review authors describe the included studies in adequate detail? (non-critical weakness);

9. Did the review authors use a satisfactory technique for assessing the risk of bias (RoB) in individual studies that were included in the review? (critical weakness);

10. Did the review authors report on the sources of funding for the studies included in the review? (non-critical weakness);

11. If meta-analysis was performed did the review authors use appropriate methods for statistical combination of results? (non-critical weakness);

12. If meta-analysis was performed, did the review authors assess the potential impact of RoB in individual studies on the results of the meta-analysis or other evidence synthesis? (non-critical weakness);

13. Did the review authors account for RoB in individual studies when interpreting/discussing the results of the review? (critical weakness);

14. Did the review authors provide a satisfactory explanation for, and discussion of, any heterogeneity observed in the results of the review? (critical weakness);

15. If they performed quantitative synthesis did the review authors carry out an adequate investigation of publication bias (small study bias) and discuss its likely impact on the results of the review? (non-critical weakness);

16. Did the review authors report any potential sources of conflict of interest, including any funding they received for conducting the review? (non-critical weakness).

References

[1] Hoy D, Brooks P, Woolf A, Blyth F, March L, Bain C, *et al*. Assessing risk of bias in prevalence studies: modification of an existing tool and evidence of interrater agreement. Journal of Clinical Epidemiology. 2012; 65: 934–939.

[2] Vandenbroucke, JP, Von Elm E, Altman DG, Gøtzsche PC, Mulrow CD, Pocock SJ, *et al*. Strengthening the reporting of observational studies in epidemiology (STROBE): explanation and elaboration. PLOS Medicine. 2007; 4: e297.
